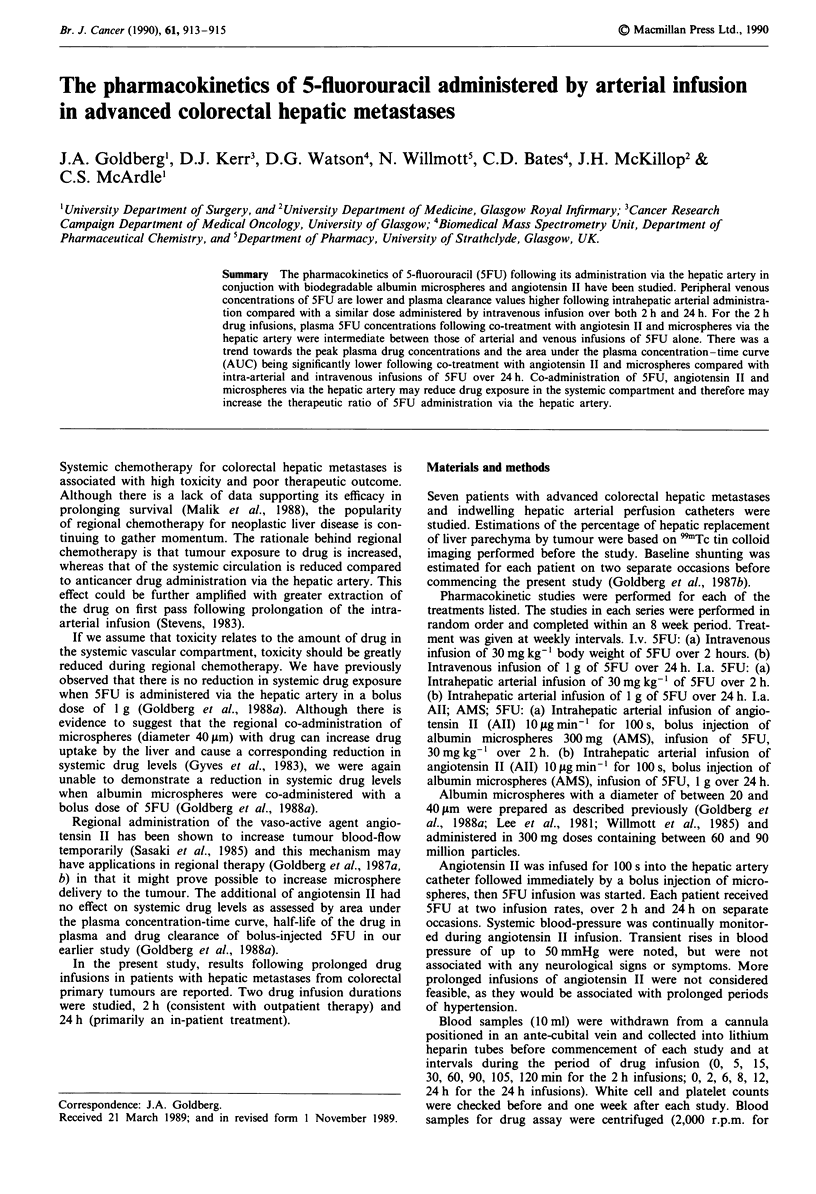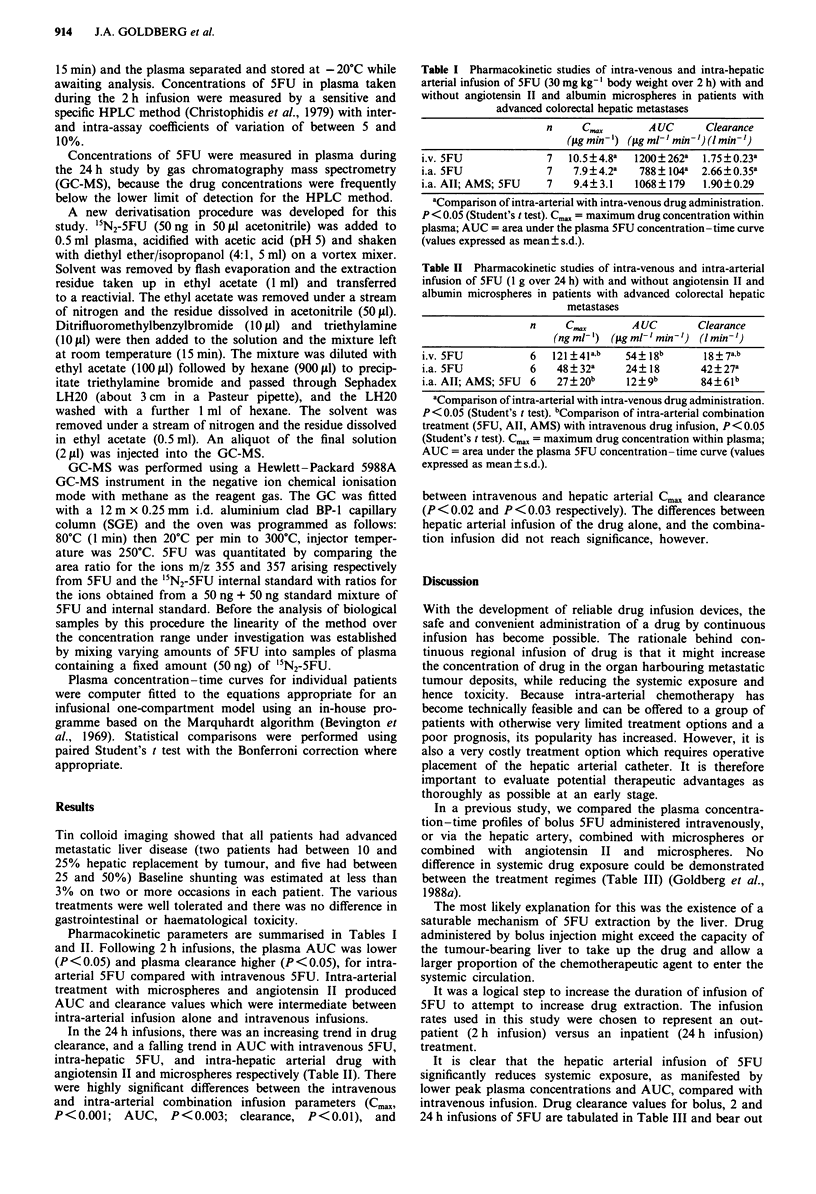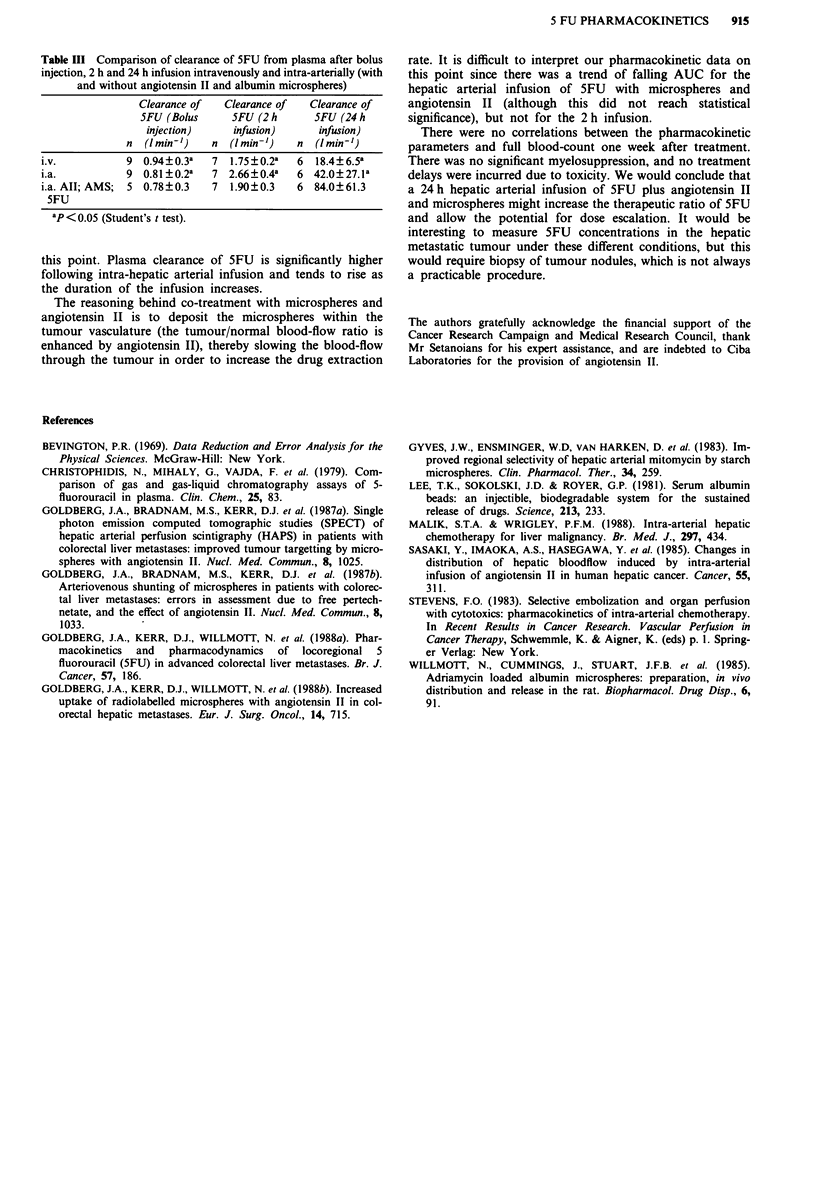# The pharmacokinetics of 5-fluorouracil administered by arterial infusion in advanced colorectal hepatic metastases.

**DOI:** 10.1038/bjc.1990.204

**Published:** 1990-06

**Authors:** J. A. Goldberg, D. J. Kerr, D. G. Watson, N. Willmott, C. D. Bates, J. H. McKillop, C. S. McArdle

**Affiliations:** University Department of Surgery, Glasgow Royal Infirmary, UK.

## Abstract

The pharmacokinetics of 5-fluorouracil (5FU) following its administration via the hepatic artery in conjunction with biodegradable albumin microspheres and angiotensin II have been studied. Peripheral venous concentrations of 5FU are lower and plasma clearance values higher following intrahepatic arterial administration compared with a similar dose administered by intravenous infusion over both 2 h and 24 h. For the 2 h drug infusions, plasma 5FU concentrations following co-treatment with angiotensin II and microspheres via the hepatic artery were intermediate between those of arterial and venous infusions of 5FU alone. There was a trend towards the peak plasma drug concentrations and the area under the plasma concentration-time curve (AUC) being significantly lower following co-treatment with angiotensin II and microspheres compared with intra-arterial and intravenous infusions of 5FU over 24 h. Co-administration of 5FU, angiotensin II and microspheres via the hepatic artery may reduce drug exposure in the systemic compartment and therefore may increase the therapeutic ratio of 5FU administration via the hepatic artery.


Br. J. Cancer (1990), 61, 913-915 © Macmillan Press Ltd., 1990

### The pharmacokinetics of 5-fluorouracil administered by arterial infusion in advanced colorectal hepatic metastases

J.A. Goldberg[1], D.J. Kerr[3], D.G. Watson[4], N. Willmott[5], C.D. Bates[4], J.H. McKillop[2] & C.S. McArdle[1]

[1]*University Department of Surgery, and* [2]*University Department of Medicine, Glasgow Royal Infirmary;* [3]*Cancer Research Campaign Department of Medical Oncology, University of Glasgow;* [4]*Biomedical Mass Spectrometry Unit, Department of Pharmaceutical Chemistry, and* [5]*Department of Pharmacy, University of Strathclyde, Glasgow, UK.*

**Summary** The pharmacokinetics of 5-fluorouracil (5FU) following its administration via the hepatic artery in conjuction with biodegradable albumin microspheres and angiotensin II have been studied. Peripheral venous concentrations of 5FU are lower and plasma clearance values higher following intrahepatic arterial administration compared with a similar dose administered by intravenous infusion over both 2 h and 24 h. For the 2 h drug infusions, plasma 5FU concentrations following co-treatment with angiotesin II and microspheres via the hepatic artery were intermediate between those of arterial and venous infusions of 5FU alone. There was a trend towards the peak plasma drug concentrations and the area under the plasma concentration–time curve (AUC) being significantly lower following co-treatment with angiotensin II and microspheres compared with intra-arterial and intravenous infusions of 5FU over 24 h. Co-administration of 5FU, angiotensin II and microspheres via the hepatic artery may reduce drug exposure in the systemic compartment and therefore may increase the therapeutic ratio of 5FU administration via the hepatic artery.

Systemic chemotherapy for colorectal hepatic metastases is associated with high toxicity and poor therapeutic outcome. Although there is a lack of data supporting its efficacy in prolonging survival (Malik *et al.*, 1988), the popularity of regional chemotherapy for neoplastic liver disease is continuing to gather momentum. The rationale behind regional chemotherapy is that tumour exposure to drug is increased, whereas that of the systemic circulation is reduced compared to anticancer drug administration via the hepatic artery. This effect could be further amplified with greater extraction of the drug on first pass following prolongation of the intra-arterial infusion (Stevens, 1983).

If we assume that toxicity relates to the amount of drug in the systemic vascular compartment, toxicity should be greatly reduced during regional chemotherapy. We have previously observed that there is no reduction in systemic drug exposure when 5FU is administered via the hepatic artery in a bolus dose of 1 g (Goldberg *et al.*, 1988*a*). Although there is evidence to suggest that the regional co-administration of microspheres (diameter 40 μm) with drug can increase drug uptake by the liver and cause a corresponding reduction in systemic drug levels (Gyves *et al.*, 1983), we were again unable to demonstrate a reduction in systemic drug levels when albumin microspheres were co-administered with a bolus dose of 5FU (Goldberg *et al.*, 1988*a*).

Regional administration of the vaso-active agent angiotensin II has been shown to increase tumour blood-flow temporarily (Sasaki *et al.*, 1985) and this mechanism may have applications in regional therapy (Goldberg *et al.*, 1987*a*, *b*) in that it might prove possible to increase microsphere delivery to the tumour. The additional of angiotensin II had no effect on systemic drug levels as assessed by area under the plasma concentration-time curve, half-life of the drug in plasma and drug clearance of bolus-injected 5FU in our earlier study (Goldberg *et al.*, 1988*a*).

In the present study, results following prolonged drug infusions in patients with hepatic metastases from colorectal primary tumours are reported. Two drug infusion durations were studied, 2 h (consistent with outpatient therapy) and 24 h (primarily an in-patient treatment).

Correspondence: J.A. Goldberg.
Received 21 March 1989; and in revised form 1 November 1989.

### Materials and methods

Seven patients with advanced colorectal hepatic metastases and indwelling hepatic arterial perfusion catheters were studied. Estimations of the percentage of hepatic replacement of liver parechyma by tumour were based on $^{99m}$Tc tin colloid imaging performed before the study. Baseline shunting was estimated for each patient on two separate occasions before commencing the present study (Goldberg *et al.*, 1987*b*).

Pharmacokinetic studies were performed for each of the treatments listed. The studies in each series were performed in random order and completed within an 8 week period. Treatment was given at weekly intervals. I.v. 5FU: (a) Intravenous infusion of 30 mg kg$^{-1}$ body weight of 5FU over 2 hours. (b) Intravenous infusion of 1 g of 5FU over 24 h. I.a. 5FU: (a) Intrahepatic arterial infusion of 30 mg kg$^{-1}$ of 5FU over 2 h. (b) Intrahepatic arterial infusion of 1 g of 5FU over 24 h. I.a. AII; AMS; 5FU: (a) Intrahepatic arterial infusion of angiotensin II (AII) 10 μg min$^{-1}$ for 100 s, bolus injection of albumin microspheres 300 mg (AMS), infusion of 5FU, 30 mg kg$^{-1}$ over 2 h. (b) Intrahepatic arterial infusion of angiotensin II (AII) 10 μg min$^{-1}$ for 100 s, bolus injection of albumin microspheres (AMS), infusion of 5FU, 1 g over 24 h.

Albumin microspheres with a diameter of between 20 and 40 μm were prepared as described previously (Goldberg *et al.*, 1988*a*; Lee *et al.*, 1981; Willmott *et al.*, 1985) and administered in 300 mg doses containing between 60 and 90 million particles.

Angiotensin II was infused for 100 s into the hepatic artery catheter followed immediately by a bolus injection of microspheres, then 5FU infusion was started. Each patient received 5FU at two infusion rates, over 2 h and 24 h on separate occasions. Systemic blood-pressure was continually monitored during angiotensin II infusion. Transient rises in blood pressure of up to 50 mmHg were noted, but were not associated with any neurological signs or symptoms. More prolonged infusions of angiotensin II were not considered feasible, as they would be associated with prolonged periods of hypertension.

Blood samples (10 ml) were withdrawn from a cannula positioned in an ante-cubital vein and collected into lithium heparin tubes before commencement of each study and at intervals during the period of drug infusion (0, 5, 15, 30, 60, 90, 105, 120 min for the 2 h infusions; 0, 2, 6, 8, 12, 24 h for the 24 h infusions). White cell and platelet counts were checked before and one week after each study. Blood samples for drug assay were centrifuged (2,000 r.p.m. for

15 min) and the plasma separated and stored at −20°C while awaiting analysis. Concentrations of 5FU in plasma taken during the 2 h infusion were measured by a sensitive and specific HPLC method (Christophidis et al., 1979) with inter- and intra-assay coefficients of variation of between 5 and 10%.

Concentrations of 5FU were measured in plasma during the 24 h study by gas chromatography mass spectrometry (GC-MS), because the drug concentrations were frequently below the lower limit of detection for the HPLC method.

A new derivatisation procedure was developed for this study. $^{15}N_2$-5FU (50 ng in 50 µl acetonitrile) was added to 0.5 ml plasma, acidified with acetic acid (pH 5) and shaken with diethyl ether/isopropanol (4:1, 5 ml) on a vortex mixer. Solvent was removed by flash evaporation and the extraction residue taken up in ethyl acetate (1 ml) and transferred to a reactivial. The ethyl acetate was removed under a stream of nitrogen and the residue dissolved in acetonitrile (50 µl). Ditrifluoromethylbenzylbromide (10 µl) and triethylamine (10 µl) were then added to the solution and the mixture left at room temperature (15 min). The mixture was diluted with ethyl acetate (100 µl) followed by hexane (900 µl) to precipitate triethylamine bromide and passed through Sephadex LH20 (about 3 cm in a Pasteur pipette), and the LH20 washed with a further 1 ml of hexane. The solvent was removed under a stream of nitrogen and the residue dissolved in ethyl acetate (0.5 ml). An aliquot of the final solution (2 µl) was injected into the GC-MS.

GC-MS was performed using a Hewlett-Packard 5988A GC-MS instrument in the negative ion chemical ionisation mode with methane as the reagent gas. The GC was fitted with a 12 m × 0.25 mm i.d. aluminium clad BP-1 capillary column (SGE) and the oven was programmed as follows: 80°C (1 min) then 20°C per min to 300°C, injector temperature was 250°C. 5FU was quantitated by comparing the area ratio for the ions m/z 355 and 357 arising respectively from 5FU and the $^{15}N_2$-5FU internal standard with ratios for the ions obtained from a 50 ng + 50 ng standard mixture of 5FU and internal standard. Before the analysis of biological samples by this procedure the linearity of the method over the concentration range under investigation was established by mixing varying amounts of 5FU into samples of plasma containing a fixed amount (50 ng) of $^{15}N_2$-5FU.

Plasma concentration-time curves for individual patients were computer fitted to the equations appropriate for an infusional one-compartment model using an in-house programme based on the Marquhardt algorithm (Bevington et al., 1969). Statistical comparisons were performed using paired Student's t test with the Bonferroni correction where appropriate.

### Results

Tin colloid imaging showed that all patients had advanced metastatic liver disease (two patients had between 10 and 25% hepatic replacement by tumour, and five had between 25 and 50%) Baseline shunting was estimated at less than 3% on two or more occasions in each patient. The various treatments were well tolerated and there was no difference in gastrointestinal or haematological toxicity.

Pharmacokinetic parameters are summarised in Tables I and II. Following 2 h infusions, the plasma AUC was lower ($P<0.05$) and plasma clearance higher ($P<0.05$), for intra-arterial 5FU compared with intravenous 5FU. Intra-arterial treatment with microspheres and angiotensin II produced AUC and clearance values which were intermediate between intra-arterial infusion alone and intravenous infusions.

In the 24 h infusions, there was an increasing trend in drug clearance, and a falling trend in AUC with intravenous 5FU, intra-hepatic 5FU, and intra-hepatic arterial drug with angiotensin II and microspheres respectively (Table II). There were highly significant differences between the intravenous and intra-arterial combination infusion parameters ($C_{max}$, $P<0.001$; AUC, $P<0.003$; clearance, $P<0.01$), and between intravenous and hepatic arterial $C_{max}$ and clearance ($P<0.02$ and $P<0.03$ respectively). The differences between hepatic arterial infusion of the drug alone, and the combination infusion did not reach significance, however.

**Table I** Pharmacokinetic studies of intra-venous and intra-hepatic arterial infusion of 5FU (30 mg kg$^{-1}$ body weight over 2 h) with and without angiotensin II and albumin microspheres in patients with advanced colorectal hepatic metastases

| | *n* | $C_{max}$ (µg min$^{-1}$) | *AUC* (µg ml$^{-1}$ min$^{-1}$) | *Clearance* (l min$^{-1}$) |
|---|---|---|---|---|
| i.v. 5FU | 7 | 10.5±4.8[a] | 1200±262[a] | 1.75±0.23[a] |
| i.a. 5FU | 7 | 7.9±4.2[a] | 788±104[a] | 2.66±0.35[a] |
| i.a. AII; AMS; 5FU | 7 | 9.4±3.1 | 1068±179 | 1.90±0.29 |

[a]Comparison of intra-arterial with intra-venous drug administration. $P<0.05$ (Student's t test). $C_{max}$ = maximum drug concentration within plasma; AUC = area under the plasma 5FU concentration-time curve (values expressed as mean ± s.d.).

**Table II** Pharmacokinetic studies of intra-venous and intra-arterial infusion of 5FU (1 g over 24 h) with and without angiotensin II and albumin microspheres in patients with advanced colorectal hepatic metastases

| | *n* | $C_{max}$ (ng ml$^{-1}$) | *AUC* (µg ml$^{-1}$ min$^{-1}$) | *Clearance* (l min$^{-1}$) |
|---|---|---|---|---|
| i.v. 5FU | 6 | 121±41[a,b] | 54±18[b] | 18±7[a,b] |
| i.a. 5FU | 6 | 48±32[a] | 24±18 | 42±27[a] |
| i.a. AII; AMS; 5FU | 6 | 27±20[b] | 12±9[b] | 84±61[b] |

[a]Comparison of intra-arterial with intra-venous drug administration. $P<0.05$ (Student's t test). [b]Comparison of intra-arterial combination treatment (5FU, AII, AMS) with intravenous drug infusion, $P<0.05$ (Student's t test). $C_{max}$ = maximum drug concentration within plasma; AUC = area under the plasma 5FU concentration-time curve (values expressed as mean ± s.d.).

### Discussion

With the development of reliable drug infusion devices, the safe and convenient administration of a drug by continuous infusion has become possible. The rationale behind continuous regional infusion of drug is that it might increase the concentration of drug in the organ harbouring metastatic tumour deposits, while reducing the systemic exposure and hence toxicity. Because intra-arterial chemotherapy has become technically feasible and can be offered to a group of patients with otherwise very limited treatment options and a poor prognosis, its popularity has increased. However, it is also a very costly treatment option which requires operative placement of the hepatic arterial catheter. It is therefore important to evaluate potential therapeutic advantages as thoroughly as possible at an early stage.

In a previous study, we compared the plasma concentration-time profiles of bolus 5FU administered intravenously, or via the hepatic artery, combined with microspheres or combined with angiotensin II and microspheres. No difference in systemic drug exposure could be demonstrated between the treatment regimes (Table III) (Goldberg et al., 1988a).

The most likely explanation for this was the existence of a saturable mechanism of 5FU extraction by the liver. Drug administered by bolus injection might exceed the capacity of the tumour-bearing liver to take up the drug and allow a larger proportion of the chemotherapeutic agent to enter the systemic circulation.

It was a logical step to increase the duration of infusion of 5FU to attempt to increase drug extraction. The infusion rates used in this study were chosen to represent an outpatient (2 h infusion) versus an inpatient (24 h infusion) treatment.

It is clear that the hepatic arterial infusion of 5FU significantly reduces systemic exposure, as manifested by lower peak plasma concentrations and AUC, compared with intravenous infusion. Drug clearance values for bolus, 2 and 24 h infusions of 5FU are tabulated in Table III and bear out

**Table III** Comparison of clearance of 5FU from plasma after bolus injection, 2 h and 24 h infusion intravenously and intra-arterially (with and without angiotensin II and albumin microspheres)

| | *n* | *Clearance of 5FU (Bolus injection) ($l\,min^{-1}$)* | *n* | *Clearance of 5FU (2 h infusion) ($l\,min^{-1}$)* | *n* | *Clearance of 5FU (24 h infusion) ($l\,min^{-1}$)* |
|---|---|---|---|---|---|---|
| i.v. | 9 | 0.94±0.3[a] | 7 | 1.75±0.2[a] | 6 | 18.4±6.5[a] |
| i.a. | 9 | 0.81±0.2[a] | 7 | 2.66±0.4[a] | 6 | 42.0±27.1[a] |
| i.a. AII; AMS; 5FU | 5 | 0.78±0.3 | 7 | 1.90±0.3 | 6 | 84.0±61.3 |

[a] $P<0.05$ (Student's *t* test).

this point. Plasma clearance of 5FU is significantly higher following intra-hepatic arterial infusion and tends to rise as the duration of the infusion increases.

The reasoning behind co-treatment with microspheres and angiotensin II is to deposit the microspheres within the tumour vasculature (the tumour/normal blood-flow ratio is enhanced by angiotensin II), thereby slowing the blood-flow through the tumour in order to increase the drug extraction rate. It is difficult to interpret our pharmacokinetic data on this point since there was a trend of falling AUC for the hepatic arterial infusion of 5FU with microspheres and angiotensin II (although this did not reach statistical significance), but not for the 2 h infusion.

There were no correlations between the pharmacokinetic parameters and full blood-count one week after treatment. There was no significant myelosuppression, and no treatment delays were incurred due to toxicity. We would conclude that a 24 h hepatic arterial infusion of 5FU plus angiotensin II and microspheres might increase the therapeutic ratio of 5FU and allow the potential for dose escalation. It would be interesting to measure 5FU concentrations in the hepatic metastatic tumour under these different conditions, but this would require biopsy of tumour nodules, which is not always a practicable procedure.

The authors gratefully acknowledge the financial support of the Cancer Research Campaign and Medical Research Council, thank Mr Setanoians for his expert assistance, and are indebted to Ciba Laboratories for the provision of angiotensin II.

### References

BEVINGTON, P.R. (1969). *Data Reduction and Error Analysis for the Physical Sciences*. McGraw-Hill: New York.

CHRISTOPHIDIS, N., MIHALY, G., VAJDA, F. *et al.* (1979). Comparison of gas and gas-liquid chromatography assays of 5-fluorouracil in plasma. *Clin. Chem.*, **25**, 83.

GOLDBERG, J.A., BRADNAM, M.S., KERR, D.J. *et al.* (1987*a*). Single photon emission computed tomographic studies (SPECT) of hepatic arterial perfusion scintigraphy (HAPS) in patients with colorectal liver metastases: improved tumour targetting by microspheres with angiotensin II. *Nucl. Med. Commun.*, **8**, 1025.

GOLDBERG, J.A., BRADNAM, M.S., KERR, D.J. *et al.* (1987*b*). Arteriovenous shunting of microspheres in patients with colorectal liver metastases: errors in assessment due to free pertechnetate, and the effect of angiotensin II. *Nucl. Med. Commun.*, **8**, 1033.

GOLDBERG, J.A., KERR, D.J., WILLMOTT, N. *et al.* (1988*a*). Pharmacokinetics and pharmacodynamics of locoregional 5 fluorouracil (5FU) in advanced colorectal liver metastases. *Br. J. Cancer*, **57**, 186.

GOLDBERG, J.A., KERR, D.J., WILLMOTT, N. *et al.* (1988*b*). Increased uptake of radiolabelled microspheres with angiotensin II in colorectal hepatic metastases. *Eur. J. Surg. Oncol.*, **14**, 715.

GYVES, J.W., ENSMINGER, W.D, VAN HARKEN, D. *et al.* (1983). Improved regional selectivity of hepatic arterial mitomycin by starch microspheres. *Clin. Pharmacol. Ther.*, **34**, 259.

LEE, T.K., SOKOLSKI, J.D. & ROYER, G.P. (1981). Serum albumin beads: an injectible, biodegradable system for the sustained release of drugs. *Science*, **213**, 233.

MALIK, S.T.A. & WRIGLEY, P.F.M. (1988). Intra-arterial hepatic chemotherapy for liver malignancy. *Br. Med. J.*, **297**, 434.

SASAKI, Y., IMAOKA, A.S., HASEGAWA, Y. *et al.* (1985). Changes in distribution of hepatic bloodflow induced by intra-arterial infusion of angiotensin II in human hepatic cancer. *Cancer*, **55**, 311.

STEVENS, F.O. (1983). Selective embolization and organ perfusion with cytotoxics: pharmacokinetics of intra-arterial chemotherapy. In *Recent Results in Cancer Research. Vascular Perfusion in Cancer Therapy*, Schwemmle, K. & Aigner, K. (eds) p. 1. Springer Verlag: New York.

WILLMOTT, N., CUMMINGS, J., STUART, J.F.B. *et al.* (1985). Adriamycin loaded albumin microspheres: preparation, *in vivo* distribution and release in the rat. *Biopharmacol. Drug Disp.*, **6**, 91.